# Can the use of older-generation beta-lactam antibiotics in livestock production over-select for beta-lactamases of greatest consequence for human medicine? An *in vitro* experimental model

**DOI:** 10.1371/journal.pone.0242195

**Published:** 2020-11-16

**Authors:** Olanrewaju J. Ogunrinu, Keri N. Norman, Javier Vinasco, Gizem Levent, Sara D. Lawhon, Virginia R. Fajt, Victoria V. Volkova, Tara Gaire, Toni L. Poole, Kenneth J. Genovese, Thomas E. Wittum, H. Morgan Scott

**Affiliations:** 1 Department of Veterinary Pathobiology, Texas A&M University, College Station, Texas, United States of America; 2 Department of Veterinary Integrative Biosciences, Texas A&M University, College Station, Texas, United States of America; 3 Department of Veterinary Physiology & Pharmacology, Texas A&M University, College Station, Texas, United States of America; 4 Department of Diagnostic Medicine/Pathobiology, Kansas State University, Manhattan, Kansas, United States of America; 5 Southern Plains Agricultural Research Center, United States Department of Agriculture, College Station, Texas, United States of America; 6 Department of Veterinary Preventive Medicine, The Ohio State University, Columbus, Ohio, United States of America; Nitte University, INDIA

## Abstract

Though carbapenems are not licensed for use in food animals in the U.S., carbapenem resistance among Enterobacteriaceae has been identified in farm animals and their environments. The objective of our study was to determine the extent to which older-generation β-lactam antibiotics approved for use in food animals in the U.S. might differentially select for resistance to antibiotics of critical importance to human health, such as carbapenems. *Escherichia coli (E*. *coli)* strains from humans, food animals, or the environment bearing a single β-lactamase gene (n = 20 each) for *bla*_TEM-1_, *bla*_CMY-2_, and *bla*_CTX-M-*_ or else *bla*_*KPC/IMP/NDM*_ (due to limited availability, often in combination with other *bla* genes), were identified, along with 20 *E*. *coli* strains lacking any known beta-lactamase genes. Baseline estimates of intrinsic bacterial fitness were derived from the population growth curves. Effects of ampicillin (32 μg/mL), ceftriaxone (4 μg/mL) and meropenem (4 μg/mL) on each strain and resistance-group also were assessed. Further, *in vitro* batch cultures were prepared by mixing equal concentrations of 10 representative *E*. *coli* strains (two from each resistance gene group), and each mixture was incubated at 37°C for 24 hours in non-antibiotic cation-adjusted Mueller-Hinton II (CAMH-2) broth, ampicillin + CAMH-2 broth (at 2, 4, 8, 16, and 32 μg/mL) and ceftiofur + CAMH-2 broth (at 0.5, 1, 2, 4, and 8μg/mL). Relative and absolute abundance of resistance-groups were estimated phenotypically. Line plots of the raw data were generated, and non-linear Gompertz models and multilevel mixed-effect linear regression models were fitted to the data. The observed strain growth rate distributions were significantly different across the groups. AmpC strains (i.e., *bla*_CMY-2_) had distinctly less robust (p < 0.05) growth in ceftriaxone (4 μg/mL) compared to extended-spectrum beta-lactamase (ESBL) producers harboring *bla*_CTX-M-*_variants. With increasing beta-lactam antibiotic concentrations, relative proportions of ESBLs and CREs were over-represented in the mixed bacterial communities; importantly, this was more pronounced with ceftiofur than with ampicillin. These results indicate that aminopenicillins and extended-spectrum cephalosporins would be expected to propagate carbapenemase-producing Enterobacteriaceae in food animals if and when Enterobacteriaceae from human health care settings enter the food animal environment.

## Introduction

Antimicrobial resistance (AMR) is one of the leading challenges of modern medicine [1]. Bacterial resistance to antimicrobials has been increasing over the decades with the prevalence of AMR and its rate of expansion closely tied to the cumulative quantities and categories of antimicrobial agents being used [2]. Bacterial pathogens resistant to antimicrobials can significantly increase the morbidity and mortality of the diseases caused in infected humans and animals [3]. Further, resistance to available antimicrobials can lead to limited–or even complete lack of–treatment options for use by human medical and veterinary practitioners. To extend the clinical efficacy of currently available drugs long into the future, prudent use of antimicrobials is firmly and widely encouraged to help mitigate AMR [2–4].

Beta-lactams are the most widely used group of antimicrobial agents in human bacterial disease treatment [5]. Newer beta-lactam antibiotics, including third- and fourth-generation cephalosporins (3GC and 4GC, respectively), carbapenems and monobactams, are important contributors to human medical as well as veterinary companion animal antimicrobial prescriptions; in part, this is due to their broad spectrum of activity, low toxicity, reliable effectiveness, and relative affordability [5]. For instance, a review of the Intercontinental Medical Statistics (IMS) data by Bush & Bradford (2016) showed that in a ten-year period (2004–2014) newer beta lactam antibiotics accounted for 65% of all parenteral antimicrobial prescriptions in the United States; of these, 47% were cephalosporins [6]. Depending on their molecular structure, beta-lactams can possess activity against either–or both–gram-positive and gram-negative bacteria [6]. Earlier generation penicillins and cephalosporins demonstrate activity against gram-positive bacteria. Potentiated aminopenicillins and later generations of cephalosporins show increasing gram-negative activity [7,8]. Carbapenems are the newest beta-lactams; these antibiotics, along with drugs belonging to the fourth and fifth generation of cephalosporins, are active against both gram-positive and gram-negative bacteria. The indications for beta-lactams are broad and include urinary tract infections, respiratory tract infections, peri-operative care, meningitis and septicemia [9,10]. In United States (U.S.) animal agriculture, three types of beta-lactams are approved for use: 1) various penicillin and aminopenicillin preparations, 2) cephapirin, and 3) ceftiofur (a 3GC used only in animals with chemical structure and pharmacological properties similar to the human drug ceftriaxone). Their clinical indications include treatment and control of respiratory tract infections, acute metritis, mastitis and foot rot. More recently, the use of cephalosporins–and ceftiofur in particular–has become more restricted due to a prohibition order against certain types of extra-label antibiotic use issued by the U.S. Food and Drug Administration (FDA) in 2012 [11].

Most Enterobacteriaceae, including *Escherichia coli* (*E*. *coli*), *Enterobacter* spp., *Citrobacter* spp. and *Klebsiella* spp., are normal commensals of the mammalian large intestine [12,13]. When systemic antimicrobials are administered to treat infections in one organ system (e.g., respiratory tract, urinary tract), another well-documented but unintended consequence is the direct selection of resistant organisms in the host gastrointestinal tract [14]. Consequently, Enterobacteriaceae, and particularly *E*. *coli*, are commonly used indicators of bacterial resistance prevalence reflecting the immediate as well as the cumulative effects of antibiotic selection pressures over the decades [15]. Furthermore, most of these commensal species can cause opportunistic infections when host defense mechanisms are compromised; for instance, *Enterobacter cloacae* and *Klebsiella* spp. are major causes of healthcare-associated infections (HAI–also known as nosocomial infections) [16]. The public health importance of Enterobacteriaceae and related gram-negative bacteria (e.g., pseudomonads) is further magnified by their ability to readily exchange mobile genetic elements (MGEs)–e.g., plasmids and transposons–that carry various antimicrobial resistance genes, thus spreading antimicrobial resistance from intestinal commensals to more highly pathogenic strains [17].

Beta-lactam resistance among Enterobacteriaceae is largely mediated through beta-lactamase enzyme production [18]; of importance, the initial discovery of beta-lactam inactivating enzymes preceded the clinical introduction of the first beta-lactam: penicillin [19]. Two beta-lactamase classification schemes are commonly recognized, the more widely recognized Ambler structural classification (Groups A-D) [20,21] and the Bush-Jacoby-Medeiros functional classification (Groups 1–4) [22,23]. Ambler groups A, C & D are active site serine metabolizers while group B enzymes are metallo-beta-lactamases; as such, this latter class of enzymes requires a zinc ion co-factor for hydrolytic activity [24]. Older recognized plasmid-borne beta-lactamase resistance genes of public health importance include: *bla*_TEM-1/2_ [25], *bla*_SHV-1_, and *bla*_OXA-1_ [18], all of which are active against amino-penicillins and narrow spectrum cephalosporins. The *bla*_CMY-*_ gene-bearing organisms (i.e., mobilized AmpC producers) are capable of inhibiting extended spectrum cephalosporins (3GC), cephamycins (2GC), many beta-lactam/beta-lactamase inhibitor (BLI) combinations (i.e., those with clavulanic acid or sulbactam), along with the older-generation beta-lactams [26]. Among others, enzymes encoded by *bla*_TEM-3_ and higher subscript designations, *bla*_SHV-2_ and higher subscript designations, and all of the *bla*_CTX-M-*_ variant genes hydrolyze extended and broad spectrum cephalosporins (i.e., 3GC & 4GC) and are thus known as extended-spectrum beta-lactamases (ESBL). The *bla*_KPC-*_, *bla*_IMP-*_, *bla*_VIM_, *bla*_NDM-*_, and *bla*_OXA-23+_ genes each encode carbapenemase enzyme production [27–31]. With the exception of the *bla*_OXA-48/181_-type gene carriers, carbapenemase producers are potent neutralizers of almost all beta-lactams, including carbapenems [28,29].

Antimicrobial use in animal agriculture has been reported to contribute to increased AMR prevalence, both in animals and in humans [32]. Some experts have suggested that uses in livestock production account for about two-thirds of the global antibiotic sales and consumption [33]. Although the risk posed to humans by direct acquisition of AMR organisms from food animals is difficult to quantify, ample evidence exists of human acquisition of resistant bacteria via food animal sources [34–39].

Carbapenems are the ‘last line of defense’ beta-lactams; as such, they have never been approved for use in food animals [40]. Nevertheless, carbapenemase-producing Enterobacteriaceae (CPE) have been isolated globally in a variety of food animal systems. The enzyme in *Acinetobacter baumannii* carrying *bla*_OXA-32_ was the first carbapenemase-producing bacterium identified in food animals, and was isolated in dairy cattle in France [28]. In 2011, a year after the finding in France, the German Research Network (RESET) project found *E*. *coli* and multiple *Salmonella* isolates harboring the *bla*_VIM-1_ gene in pig feces, the pig farm environment, and in poultry dust [41–43]. Other carbapenemase-producing bacteria have been found in food animals in places such as China (*A*. *baumannii*: *bla*_NDM-1_) [44,45] and Lebanon (*Pseudomonas aeruginosa*: *bla*_VIM-2_, *A*. *baumannii*: *bla*_OXA-23_) [46]. In the High Plains region of Texas and New Mexico, the first U.S. animal agricultural CPE were identified in 2016; of note, one *A*. *baumannii* from dairy cattle excreta carried a seemingly novel chromosomal gene *bla*_OXA-497_ [47]. Later, plasmid-borne *bla*_IMP-27/64_ genes were detected in multiple Enterobacteriaceae species from the environment and fecal samples of a farrow-to-finish swine operation in the Midwest region of the U.S. This latter finding included the observation that the CPE isolates were found in pig barns where a 3GC ceftiofur was used, but not in barns where its use was not recorded [48,49].

The increasing isolation of CPEs from food animal agricultural settings poses somewhat of a conundrum. Mollenkopf et al. (2016) proposed that approved beta-lactams in animal agriculture may differentially select for carbapenemase-producing strains in farm animals and their environment [48,50]. Other authors such as Woodford et al., (2014) made a similar but broader suggestion that multiple classes of antimicrobials other than the beta-lactams may provide the selection pressures for carbapenem-resistance Enterobacteriaceae (CRE) isolates in animal agriculture [51]. Multiple genes encoding resistance to different antimicrobial agents are frequently found together on mobile genetic elements–such as plasmids harbored by Enterobacteriaceae–and this can facilitate co-selection of resistance. However, no evidence exists to date to support assertions that early-generation beta-lactams might select disproportionately for higher-level beta-lactam resistance, including CPEs, in the absence of that co-selection mechanism.

The objective of this study was to determine the extent to which older-generation β-lactam antibiotics approved in the U.S. for use in food animals (e.g., aminopenicillins and 1st generation cephalosporins) can differentially select for highest priority antibiotic resistance (e.g., to 3rd and 4th generation cephalosporins and carbapenems, respectively) among representative Enterobacteriaceae. In addition to characterizing individual strain fitness in both antibiotic containing and non-antibiotic media, we also aimed to test the differential selection dynamics of a 5-group mixture of host-adapted *E*. *coli* strains; as far as possible, with each group bearing one, but not a combination, of beta-lactamase genes: *bla*_TEM-1_, *bla*_CMY-2_, *bla*_CTX-M-*_, or *bla*_*KPC/IMP/NDM*_, along with a group of beta-lactamase-free strains. Through *in vitro* experiments and statistical modeling, we aimed to narrow the existing knowledge gap concerning the potential for over-selection of ESBLs and CPEs in U.S. food animal production through approved uses of ampicillin and ceftiofur, respectively.

## Materials and methods

### Microbiologic methods

#### Bacterial strain selection

Bacteria used in this study were sourced from our strain collections (HMS and TEW) as well as from the jointly sponsored U.S. Centers for Disease Control and Prevention (CDC) and the Food and Drug Administration (FDA) Antimicrobial Resistance (AR) Isolate-Bank [52]. Isolates from our own collections were characterized as to antimicrobial resistance, both phenotypically and genotypically, in previous research projects in our (HMS and TEW) groups. Detailed methodologies employed in those characterizations have previously been published [48,49,53]. For carbapenem-producing Enterobacteriaceae (CPE), initial isolation of the bacterial strains from field samples was through supplemented MacConkey broth containing meropenem at 0.5μg/mL and zinc sulfate heptahydrate 70 μg/mL [48].

Briefly, minimum inhibitory concentrations (MIC) of antimicrobial susceptibility which are routinely monitored in Enterobacteriaceae were determined for the isolates using the broth micro-dilution method. The Sensititre^TM^ system (TREK, Thermo Scientific Microbiology, Oakwood Village, OH) was employed, using gram-negative CMV3AGNF custom panels designed for the U.S. National Antimicrobial Resistance Monitoring System (NARMS); for some isolates, extended-spectrum beta-lactamase ESB1F panels (TREK, Thermo Scientific Microbiology, Oakwood Village, OH) also were used to further characterize the beta-lactam phenotypic susceptibility (i.e., AmpC versus ESBL versus carbapenemase). Bacterial antimicrobial testing was performed in accordance with published NARMS protocols [54]. Outcomes expressed as antimicrobial minimum inhibitory concentrations (MIC) were interpreted according to the clinical interpretative human breakpoint values recommended for *E*. *coli* by the Clinical Laboratory Standards Institute (CLSI) as listed below, or else as their raw values befitting the type of the statistical analysis performed [55].

Bacterial genotypes were determined in our laboratories through short-read whole genome sequencing. In brief, bacterial genomic DNA was extracted with the QIAamp DNA extraction kit on the QIAcube HT automated platform (QIAGEN, Valencia, CA) while library preparation was with the Illumina Nextera XT or DNA Flex Kits (Illumina Inc, San Diego, CA). Sequencing runs were performed with the MiSeq Reagent Kit v3 paired-end reads (2 x 300 bp) on the Illumina MiSeq instrument (Illumina Inc., San Diego, California). Post-run bioinformatic analyses were performed on the BaseSpace Sequence Hub (Illumina Inc., San Diego, California). Depending on the source of the isolate, nucleotide sequence reads were assembled with either Velvet *de novo* or SPAdes genome assembler software [56,57]; bacteria sequence types were determined with the SRST2 Basespace application (Illumina Inc., San Diego, California). Antimicrobial resistant gene annotation was with the Antibiotic Resistance Gene-ANNOTation database (ARG-Annot) or ResFinder [58] and plasmid type annotation was via PlasmidFinder [59]. Genotypic and phenotypic characterizations of isolates obtained from the CDC/FDA AR Isolate Bank were as published on the agency website and accessible from the National Center for Biotechnology Information (NCBI) portal [52]. For the purpose of uniformity in data presentation, and to further ascertain prior strain annotations from the aforementioned sources, raw reads of strains used in this study were again, either pulled from NCBI (National Center for Biotechnology Information, U.S. National Library of Medicine) SRA (sequence read archive) [60] or from our archive. Nucleotide sequence reads were assembled using SPAdes ver.3.11.1 genome assembler softwares [57]; bacteria sequence types were determined with the SRST2ver.0.2.0 [61] and Multi-Locus Sequence Typing (MLST) database of *Escherichia coli* (accessed on May, 2020) [62]. Antimicrobial resistant gene and plasmid annotation was performed using ABRicate ver.0.8.7 [63] and ResFinder [58] and PlasmidFinder [64], databases (both accessed on May, 2020). Corresponding sequence type, genotypic and phenotypic antimicrobial resistance profile, and plasmidal information of the isolates included in this study are provided under the given bioproject and biosample accession numbers in supplemental material (S1 Table).

When possible, the choice of *E*. *coli* strains of swine origin was prioritized over other bacteria sources, swine being our exemplar food animal host. Pig being monogastric possess numerous intestinal anatomical, physiological and microbiota similarities with the human digestive tract; these similarities have informed the many use of swine in modeling human intestinal parameters [65,66]; additionally, the first description of CPEs in the United States was in a swine production facility [48]. When this was not possible, strains from other livestock, human patients, or else the environment were selected. *E*. *coli* strains were initially selected according to the presence of plasmid-borne genes encoding beta-lactamases, or else a complete lack thereof. Isolates identified from this screening process were then stratified by the presence of their beta-lactamase genes; that is, *bla*_TEM-1_ or *bla*_CMY-2_ or *bla*_CTX-M-*,_ or *bla*_*KPC/IMP/NDM*_, or none, but avoiding to the extent possible those strains with a combination of *bla* genes. Each group of the selected resistance genes corresponded to a known spectrum of hydrolytic activity against beta-lactam antibiotics.

Twenty isolates were selected per *bla*-positive group or *bla*-negative control group. Ideally, 30 strains of each group would have been the sample size to achieve near-normal log_10_ transformed distribution of target quantitative outcomes under the Central Limit Theorem [67,68]; however, strains with only a single *bla* gene were limited in the sources accessible to us. Even more so for the CPEs; consequently, presence of a single *bla* gene was achieved only for ten percent of the carbapenemase producing strains (see S1 Table). Study strains were allowed to possess genes encoding resistance to other classes of antimicrobials. These genes were not expected to impact the beta-lactam antimicrobial resistance of the strains in the absence of co-selection pressures; for example, genes conferring tetracycline, aminoglycoside and sulfonamide resistance were commonly identified among all strain groups.

#### Bacterial growth curve estimation

To assess the growth rates of each bacterial strain absent and under different antibiotics and their concentrations, and to estimate the within- and among-*bla*-group growth fitness parameter differences, bacterial growth curves were estimated with the Bioscreen C^**TM**^ Automated Microbiology Growth Curve Analysis System (Growth Curve Ltd, Helsinki, Finland). Bacteria from pure culture and preserved on cryobeads at -80°C were streaked onto Trypticase™ soy agar with 5% sheep blood (Becton, Dickinson and Company, Sparks, MD) and incubated at 37°C overnight. A 0.5 McFarland standard (Sensititre^**TM**^ Thermo Fisher Scientific, Waltham, MA) bacterial suspension was made for each isolate (i.e., to a bacterial concentration of ~1.5 x 10^**8**^ CFU/mL), by suspending one or two colonies selected from the overnight plate growth in demineralized water (Thermo Fisher Scientific, Waltham, MA). A 1:10 mixture of the bacterial suspension (120 ul) in cation-adjusted Mueller-Hinton II (CAMH-2) broth (Thermo Fisher Scientific, Waltham, MA) (1,080 ul) in a 1.5 mL black sample tube was then made (final bacterial concentration of ~1.5 x 10^**7**^ CFU/mL). From this bulk mixture, 300ul aliquots in triplicate were dispensed into each 10*10 honeycomb plate well (Growth Curves USA, Piscataway, New Jersey, USA) for each strain; thereafter, automated optical density (OD) estimates were obtained at 420-580nm (wideband) over 48 hours at 37°C. Measurements (OD) were taken every 10 minutes following moderate agitation of the incubating cultures. For each experiment, a single QC strain *E*. *coli* ATCC 25922 (American Type Culture Collection, Manassas, VA) in triplicate wells, and two wells with plain CAMH-2 broth, were included as positive and negative controls, respectively.

The effects of ampicillin (an aminopenicillin), ceftriaxone (a third-generation cephalosporin (3GC)), and meropenem (a carbapenem) on the growth parameters of each strain in the corresponding *bla*-groups were estimated. The tested concentration of each antimicrobial corresponded to the human clinical resistance breakpoint value for the drug-bacteria species MIC as recommended by the CLSI [55]. Specifically, the concentrations were 32 μg/mL of ampicillin sodium, 4 μg/mL of ceftriaxone disodium, or 4μg/mL of meropenem trihydrate; high purity forms of the antimicrobials (Sigma-Aldrich Inc., St. Louis, MO) dissolved in CAMH-2 broth were used to prepare the tested concentrations. Ceftriaxone (a commonly used 3GC in human medicine) was chosen for this *in vitro* assay to represent potential selection risks in the human host.

#### Bacterial competition assay

From each resistance group (i.e., no *bla* genes,–but with a tetracycline resistance gene, *tet*(B)–*bla*_**TEM-1**_, *bla*_**CMY-2**_, *bla*_CTX-M-*_, or *bla*_**KPC/IMP/NDM**_), two representative strains were selected for competition studies. From the WGS data, isolates with the least number of co-resistance genes were selected as representative strains per strain-group to control for potential fitness constraint imposition, while equally adjusting for antimicrobial co-selection potential. A 0.5 McFarland (Sensititre^**TM**^ Thermo Fisher Scientific, Waltham, MA) standardized bacterial suspension was prepared for each selected isolate as described above. An equal mixture of all ten strains (1 mL of each suspension) was prepared (expected density of each strain in the mixture was ~1.5 x 10^**7**^ CFU/mL). A 1:10 dilution of the mixture in CAMH-2 broth, as well as in CAMH-2 broth with various antibiotic concentrations (expected density of each strain ~1.5 x 10^**6**^ CFU/mL) were made. The antimicrobial concentrations were ampicillin sodium (Sigma-Aldrich, St. Louis, MO) at 2, 4, 8, 16 or 32 μg/mL and ceftiofur hydrochloride (Zoetis Inc., Parsippany-Troy Hills, NJ) at 0.5, 1, 2, 4, 8 μg/mL. Ceftiofur was chosen for this assay to represent the 3GC used in veterinary medicine; that is, to directly mimic the selection pressure occurring *in vivo* in farm animals.

Triplicates of the mixed cultures were incubated at 37°C for 24 hours in the Bioscreen C^TM^ Automated Microbiology Growth Curve Analysis System, as previously outlined. The post-growth density of each strain group was estimated phenotypically by the spiral-plate method (Eddy Jet 2™ Spiral-Plater, Neutec Group Inc., NY); to achieve this, after 24 hours of incubation the mixed cultures were spiral-plated onto MacConkey agar plates infused with one of tetracycline—16 μg/mL, ampicillin—32 μg/mL, cefoxitin—32 μg/mL, ceftriaxone—4 μg/mL, ceftriaxone—4 μg/mL with clavulanate—4 μg/mL, cefepime—8 μg/mL, or meropenem—4 μg/mL [55]. The bacterial culture dilutions for spiral-plating were performed as necessary on ice beds (to halt bacterial growth). The agar plates were read on an automated colony counter (Flash & Go^TM^, Neutec Group Inc., NY) to obtain estimates of the bacterial density in colony forming units (CFU)/mL of the mixed culture as of 24 hours of incubation. The strain group(s) whose density was assessed using each of the selective agars is listed in Table 1. A fresh preparation of experimental bacteria mixture was also plated as outlined above.

**Table 1:** Antibiotic supplemented media with presumed *E*. *coli* genotypes and phenotypes selectively grown on each type.

MacConkey agar with antibiotic plates^a^	*E*. *coli* resistance genotype expected to grow on the plates	*E*. *coli* resistance phenotype expected to grow on the plates
Tetracycline (16 μg/mL)–(MAC+TET)	*tet*(B)	All tetracycline resistant strains
Ampicillin (32 μg/mL)–(MAC+AMP)	*bla*_TEM-1_, *bla*_CMY-2,_ *bla*_CTX-M,_ *bla*_KPC/IMP/NDM_	All beta-lactamase producing strain
Cefoxitin (32 μg/mL)–(MAC+FOX)	*bla*_CMY-2,_ *bla*_KPC/IMP/NDM_	AmpC + CPE
Ceftriaxone + clavulanic acid (4 μg/mL + 4 μg/mL)–(MAC+AXOCLAV)	*bla*_CMY-2,_ *bla*_KPC/IMP/NDM_	AmpC + CPE
Ceftriaxone (4 μg/mL)–(MAC+AXO)	*bla*_CMY-2,_ *bla*_CTX-M,_ *bla*_KPC/IMP/NDM_	AmpC + ESBL + CPE
Cefepime (8 μg/mL)–(MAC+PIME)	*bla*_CMY-2,_ *bla*_CTX-M,_ *bla*_KPC/IMP/NDM_	ESBL + CPE
Meropenem (4 μg/mL)–(MAC+MERO)	*bla*_KPC/IMP/NDM_	CPE

AmpC, molecular class C-type beta-lactamase; ESBL, extended spectrum beta-lactamase; CPE, carbapenemase-producing Enterobacteriaceae.

^a^The antibiotic concentrations correspond to human clinical interpretive breakpoints, recommended by the CLSI as of the date of experimentation [55].

### Statistical methods

#### Growth curve analyses

Data from the EZexperiment™ software (Growth Curves Ltd, Helsinki, Finland) were transformed from wide to long format based on the time of incubation (i.e., in 10-minute intervals from time = 0) in Stata version 15.1 software (Stata Corp, College Station, TX). Triplicate OD measurements were collapsed to their mean by strain at each time point to obtain a single representative value. Raw and mean measured OD of each bacterial strain was plotted against time. The isolates were stratified by resistance group and the maximal bacterial population growth rates were estimated, as well as the growth rates in presence of each of the antibiotic concentrations.

To more precisely estimate the growth parameters, four nonlinear regression models were fitted to the OD-based growth curve of the bacterial groups grown in non-selective media, using the least squares method implemented in the Stata^®^ 15.1 software (Stata Corp., College Station, TX). The four models were: 3-parameter Gompertz, 4-parameter Gompertz [69], 3-parameter logistic [70] and 3-parameter exponential [71]. Further, the relative fit of the models across all the bacterial resistance groups and experimental conditions (i.e., with and without antibiotics) was explored. The 3-parameter Gompertz (Eq 1) was found to most consistently provide the best fit (highest adjusted coefficient of determination (*R*^2^)) for the growth curves, across all antibiotic concentration/bacteria-group combinations. Consequently, the bacterial growth rate (OD/hour) estimates, after transforming time units from minutes to hours, were extracted from the 3-parameter Gompertz models fitted to these growth curves:

$$OD=\beta_{1}*e^{\left({-e}^{\left(-\beta_{2}*(t-\beta_{3})\right)}\right)}$$ (1)

Where, *t* is time in hours, *β*_*1*_ is the peak bacterial density (OD), *β*_*2*_ is the estimated maximum growth rate (Δ OD per hour) and *β*_*3*_ is the estimated bacterial growth lag period (in hours). Models were stratified by resistance gene group and the type of growth media (i.e., without and with different antibiotics at breakpoint concentrations). To obtain graphical representations of modeled growth rates, post-analysis non-linear OD marginal predictions were generated. Predicted OD were subsequently graphed against time in hours.

Strain group growth in the mixed cultures. Mixed-effect nonlinear regression models were fitted to the estimated density of each resistance group at 24 hours of incubation of the mixed-strain culture (the CFU/mL readings from the selective antibiotic MAC plates were log_**10**_ transformed to normalize the data prior to the analysis), Modeled fixed effects were the antibiotic type, the various concentrations of each antibiotic used in the growth media for the mixed culture, and the selective antimicrobial plate type. The experimental replicate was modeled as a random effect factor. Marginal mean predictions of the strain group densities from the model output with 95% confidence intervals were determined and represented graphically. The analysis also was performed in Stata^**®**^ version 15.1 software (Stata Corp., College Station, TX).

## Results

### Strain group fitness

**Fig 1:**
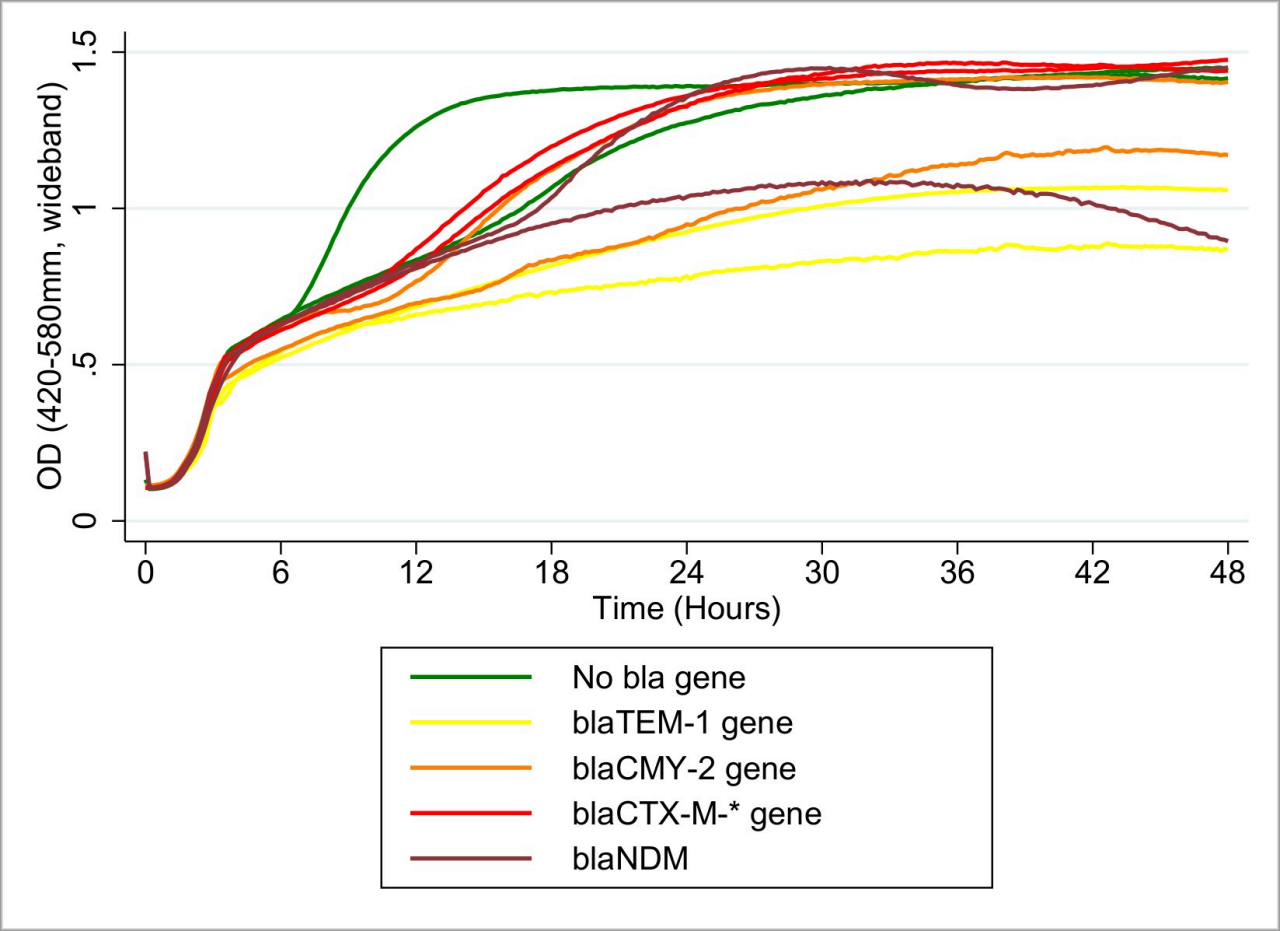
Raw optical density line plots for 10 individual strains^a^ grown in cation-adjusted Mueller-Hinton II broth. (Green) *E*. *coli* strains lacking any beta-lactamase encoding genes, (Yellow) *bla*_TEM-1_ beta-lactamase encoding gene strains, (Orange) *bla*_CMY-2_ beta-lactamase encoding gene strains, (Red) *bla*_CTX-M-*_ beta-lactamase encoding gene strains, and (Maroon) *bla*_NDM/IMP/KPC_ carbapenemase encoding gene *E*. *coli* strains. ^a^These same two selected strains per *bla*-gene group (color) were used in the multi-strain mixed-culture batch growth experiments.

In all strain groups, numerous strains demonstrated biphasic exponential growth phase–suggesting a switch in energy source utilization from glucose to a different compound [72–74] when nutrient supplies became limited–while others did not (Fig 1). All groups exhibited a lag period prior to the exponential amplification of the OD value. Within-group similarity in the first phase of exponential growth was observed for strains with and without a diauxic growth pattern. The onset of the second phase of exponential growth–when present–exhibited within- and among-group variation, as seen in Fig 1 with selected representative strains. The maximum OD value attained by any strain across all resistance gene groups was roughly the same (≈1.4), but the time taken to reach this point differed within and among groups.

**Fig 2:**
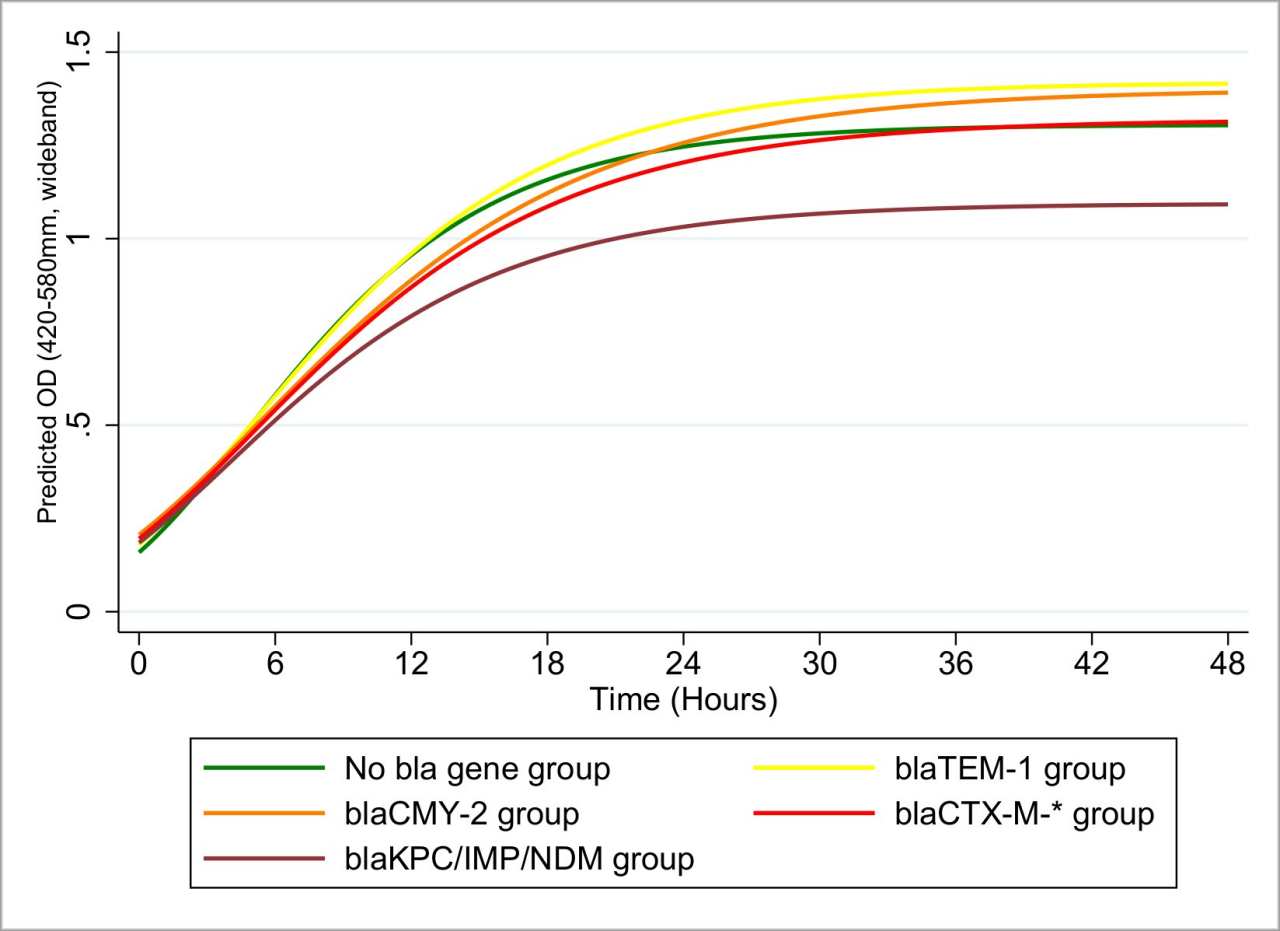
*E*. *coli* strains (n = 20 / *bla*-gene group) 3-Parameter Gompertz fitted growth curves grown in CAMH-2 broth. CAMH-2 (Cation-adjusted Mueller-Hinton II). (Green) *E*. *coli* strains lacking any beta-lactamase encoding genes, (Yellow) *bla*_TEM-1_ beta-lactamase encoding gene strains, (Orange) *bla*_CMY-2_ beta-lactamase encoding gene strains, (Red) *bla*_CTX-M-*_ beta-lactamase encoding gene strains, and (Maroon) *bla*_NDM/IMP/KPC_ carbapenemase encoding gene *E*. *coli* strains.

The 4-parameter Gompertz and the 3-parameter exponential models showed the lowest *R*^2^ values when fitted to the data (0.34 to 0.84 and 0.34 to 0.85 across the strains, respectively). The 3-parameter logistic (0.85–0.93) and 3-parameter Gompertz (0.86–0.93) models demonstrated the highest *R*^2^ values. Thus, the 3-parameter Gompertz model was fitted to all the growth curves, and the model predictions were plotted and evaluated. When modeled in the absence of beta-lactam antibiotics, the estimated peak growth rate of the beta-lactam susceptible group [0.159 (OD/hour), 95% CI: 0.152–0.166] was significantly higher than for the *bla*-positive groups (see Table 2 and Fig 2). Among the *bla* producers, the estimated growth rate of the carbapenemase producing *E*. *coli* in non-antibiotic media was highest [0.142 (OD/hour), 95% CI: 0.136–0.149]; however, this estimate was not statistically significantly different from that of the TEM-type beta-lactamase group [0.139 (OD/hour), 95% CI: 0.135–0.143], as indicated by the overlap of their respective 95% CIs. The AmpC-type beta-lactamase group and the ESBL producers exhibited statistically similar peak growth rate estimates [0.120 (OD/hour), 95% CI: 0.116–0.124] and [0.127 (OD/hour), 95% CI: 0.121–0.132]. Although the beta-lactam susceptible group showed a higher growth rate, among the beta-lactam resistant groups no fitness cost pattern along the gradient of the encoded resistance (from non-extended-to-extended or from extended-to-non-extended beta-lactam resistance) was observed.

**Table 2:** Bacterial growth curve parameter values with 95% confidence intervals as estimated by a 3-parameter Gompertz non-linear model, across resistance gene group and by antibiotic type.

			*NBL			*TEM-1			*CMY-2			*CTX-M-*			*CPE	
Growth Media		*coeff*	*95% CI*		*coeff*	*95% CI*		*Coeff*	*95% CI*		*coeff*	*95% CI*		*coeff*	*95% CI*	
			Lower	Upper		Lower	Upper		Lower	Upper		Lower	Upper		Lower	Upper
	^a^**b1**	1.305	1.296	1.315	1.419	1.412	1.426	1.400	1.391	1.409	1.318	1.307	1.329	1.094	1.085	1.103
**No Antibiotic**	^b^**b2**	0.159	0.152	0.166	0.139	0.135	0.143	0.120	0.116	0.124	0.127	0.121	0.132	0.142	0.136	0.149
	^c^**b3**	4.676	4.487	4.865	5.225	5.099	5.352	5.414	5.261	5.567	5.089	4.876	5.302	4.043	3.828	4.257
	**b1**				1.278	1.262	1.294	1.365	1.356	1.374	1.214	1.199	1.229	1.081	1.071	1.123
**Ampicillin (32 μg/mL)**	**b2**				0.144	0.135	0.154	0.122	0.118	0.125	0.125	0.117	0.133	0.142	0.135	0.155
	**b3**				5.289	4.980	5.598	5.629	5.471	5.786	4.967	4.648	5.286	3.935	3.693	4.176
	**b1**							1.011	0.968	1.054	1.161	1.143	1.179	1.087	1.077	1.096
**Ceftriaxone (4 μg/mL)**	**b2**							0.059	0.052	0.066	0.133	0.122	0.144	0.135	0.128	0.142
	**b3**							5.608	4.784	6.432	4.703	4.303	5.102	4.056	3.809	4.303
	**b1**													1.024	1.002	1.045
**Meropenem (4 μg/mL)**	**b2**													0.083	0.078	0.089
	**b3**													8.944	8.523	9.365

Where growth parameters were not estimable using the models, such as for gene groups susceptible to the antibiotic in question, results are represented as missing data.

^a^Peak bacterial density (OD at 420-580mm, wideband).

^b^Estimated growth rate (OD/hour).

^c^Estimated growth lag (hours).

*NBL–No beta-lactamase gene present, TEM-1 –*bla*_TEM-1_ gene present, CMY-2 –*bla*_CMY-2_ gene present, CTX-M-*–*bla*_CTX-M-*_ gene present, CPE–carbapenemase-producing Enterobacteriaceae gene present (e.g., *bla*_KPC_, *bla*_NDM_, *bla*_IMP_).

In a similar fashion, the combined growth rates of the representative pair of strains of each group used in the mixed cultures were predicted (Table 3). Select representative raw data line plots of OD-based growth curves from cation-adjusted Mueller-Hinton broth for 10 study strains (color-coded by the 5 resistance gene groups) are shown in Fig 1. These estimates show a 95% CI overlap among multiple group-based strain pairs; the pair of beta-lactam susceptible strains demonstrated the highest predicted growth rates [0.16 (OD/hour), 95% CI: 0.15–0.17], an estimate non-statistically different from that of the carbapenemase producers [0.13 (OD/hour), 95% CI: 0.12–0.15]. The predicted growth rates of the ESBL-producing pair [0.12 (OD/hour), 95% CI: 0.12–0.12] were observed to be non-statistically different from that of the carbapenemase producers but statistically different from the beta-lactam susceptible strains. The lowest growth rate estimates were those of the *bla*_TEM-1_ encoding strain pair [0.10 (OD/hour), 95% CI: 0.08–0.11], and the AmpC-type beta-lactamase encoding strain pair [0.10 (OD/hour), 95% CI: 0.09–0.11]. In general, the maximum growth rates of the representative pairs were not far removed from one another, suggesting a relatively similar intrinsic fitness absent an extrinsic antibiotic pressure. Notably, the estimated maximum bacterial density (peak OD) attained by the pairs appeared to differ across the groups. The ESBL-producers showed a significantly higher upper asymptote [1.49 (OD), 95% CI: 1.48–1.50], as did the beta-lactam susceptible strains [1.41 (OD), 95% CI: 1.40–1.43]. In decreasing order, the peak density estimates for the AmpC-type beta-lactamase pair, the carbapenemase producing pair and the *bla*_TEM-1_ encoding pair were: 1.34 (OD), 95% CI: 1.31–1.37, 1.24 (OD), 95% CI: 1.22–1.27, and 1.15 (OD), 95% CI: 1.11–1.19, respectively.

**Table 3:** Bacterial growth parameter values with 95% confidence intervals estimated by a 3-parameter Gompertz model, for strain pairs used in the batch competition assay.

bla *gene group*^*1*^	*Predicted growth rate (OD/hour)*	*95% CI*	*Estimated lag (hour)*	*95% CI*	*Peak density (OD)*	*95% CI*
		Lower; Upper		Lower; Upper		Lower; Upper
**NBL**	0.16	0.15; 0.17	4.79	4.55; 5.02	1.41	1.40; 1.43
**TEM-1**	0.10	0.08; 0.11	4.50	3.67; 5.34	1.15	1.11; 1.19
**CMY-2**	0.10	0.09; 0.11	5.82	5.33; 6.30	1.34	1.31; 1.37
CTX-M-*	0.12	0.12; 0.12	6.09	5.96; 6.25	1.49	1.48; 1.50
**NDM**	0.13	0.12; 0.15	4.56	4.09; 5.04	1.24	1.22; 1.27

*NBL–No beta-lactamase gene present, TEM-1 –*bla*_TEM-1_ gene present, CMY-2 –*bla*_CMY-2_ gene present, CTX-M-*–*bla*_CTX-M-*_ gene present, NDM–carbapenemase-producing Enterobacteriaceae gene present (i.e., *bla*_NDM_).

### Effect of beta-lactam antibiotics on bacterial growth rates

**Fig 3:**
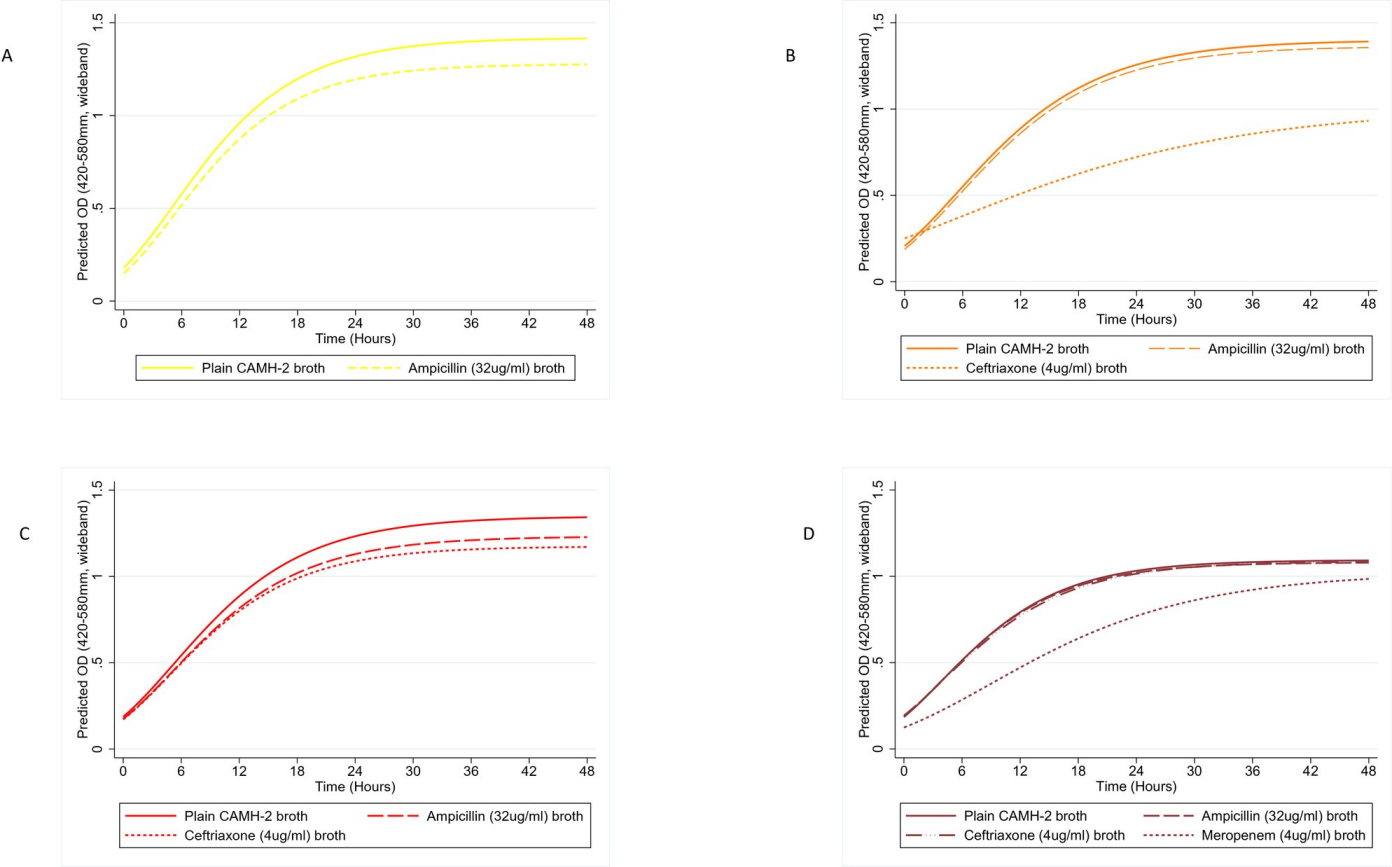
Growth curves of *bla*-gene positive *E*. *coli* resistance gene groups grown in beta-lactam antimicrobial broth, estimated with a 3-parameter Gompertz model. (a) *bla*_TEM-1_ gene encoding strains in plain CAMH-2 broth and ampicillin (32 μg/mL) broth, (b) *bla*_CMY-2_ gene encoding strains in plain CAMH-2 broth, ampicillin (32 μg/mL) and ceftriaxone (4 μg/mL) broths, (c) *bla*_CTX-M-*_ gene encoding strains in plain CAMH-2 broth, ampicillin (32 μg/mL) and ceftriaxone (4 μg/mL) broths, (d) *bla*_KPC/IMP/NDM_ encoding strains in plain CAMH-2 broth, ampicillin (32 μg/mL), ceftriaxone (4 μg/mL) and meropenem (4 μg/mL) broths. CAMH-2 (Cation-adjusted Mueller Hinton II).

The relative growth rates of the resistance-gene groups in the presence of beta-lactams of different generations (i.e., at concentrations corresponding to the human clinical interpretive breakpoints for MICs of these drugs for *E*. *coli*) were assessed, to predict preferential selection by the antibiotics for the strains with studied resistance gene groups. As expected, the beta-lactamase-free strains registered no discernable growth in media with any of the three tested beta-lactam antibiotics. All strains from the four beta-lactam resistance gene groups showed no significant growth impairment in 32 μg/mL ampicillin (though with substantive 95% CI overlap of ampicillin-containing versus plain CAMH-2 broth growth rates, respectively: [0.139 (OD/hour), 95% CI: 0.135–0.143] compared to [0.144 (OD/hour), 95% CI: 0.135–0.154] for the TEM-1 beta-lactamase group; [0.120 (OD/hour), 95% CI: 0.116–0.124] compared to [0.122 (OD/hour), 95% CI: 0.118–0.125] for the AmpC-type beta-lactamase group; [0.127 (OD/hour), 95% CI: 0.121–0.132] compared to [0.125 (OD/hour), 95% CI: 0.117–0.133] for the ESBL group; and [0.142 (OD/hour), 95% CI: 0.136–0.149]compared to [0.142 (OD/hour), 95% CI: 0.135–0.155] for the CPE group (see Table 2 and Fig 3).

Correspondingly, the CPE group and the ESBL producers both demonstrated comparable robust growth in 4 μg/mL ceftriaxone and in plain CAMH-2 broth [0.127 (OD/hour), 95% CI: 0.121–0.132] compared to [0.133 (OD/hour), 95% CI: 0.122–0.144] for ESBL producers; and [0.142 (OD/hour), 95% CI: 0.136–0.149] compared to [0.135 (OD/hour), 95% CI: 0.128–0.142] for the CPE group; however, AmpC producers (i.e., harboring only *bla*_CMY-2_) showed significantly reduced growth rates in the ceftriaxone-containing media. Comparing growth rates of the AmpC-type beta-lactamase group in plain CAMH-2 versus 4 μg/mL ceftriaxone broth, the predicted maximum growth rate dropped from 0.120 (OD/hour), 95% CI: 0.116–0.124 to 0.059 (OD/hour), 95% CI: 0.052–0.066 (see Table 2 and Fig 3). As expected, only CPE strains registered growth in 4 μg/mL meropenem; however, the presence of the drug at this concentration significantly altered the estimated peak growth rate of the strains from 0.142 (OD/hour), 95% CI: 0.136–0.149 to 0.083 (OD/hour), 95% CI: 0.078–0.089. The estimated duration of the lag phase of bacterial population growth in that media was also considerably prolonged [8.944 (hours), 95% CI: 8.523–9.365] when compared with such estimates in other tested beta-lactams (Table 2), as was the peak OD value, which was significantly reduced to 1.024 (OD), 95% CI: 1.002–1.045 from 1.094 (OD), 95% CI: 1.085–1.103 (Table 2 and Fig 3D).

### Effect of beta-lactam antibiotics on mixed bacterial populations

Initial resistance-gene group populations grown on the selective antimicrobial MAC plates (Table 1) using the starting mixture of select representative strains (see Table 1 for legend) provided CFU estimates for MAC+TET, MAC+AMP, MAC+FOX, MAC+AXO, MAC+AXOCLAV, MAC+PIME and MAC+MERO plates equivalent to 91.5%, 95.3%, 19.4%, 64.7%, 37.8%, 14.7%, and 10.7%, respectively, of the total CFU estimate from the non-selective MAC plate (Table 4). Assuming a simple substitution model with no compensatory growth through to nutrient capacity, and no negative antibiotic effects on growth of resistant strains, the expected proportion of the CFU estimate on each plate type–relative to those on the plain MAC–would have been: MAC+TET plate (~ 100%), the MAC+AMP plate (~ 80%), MAC+FOX (~40%), MAC+AXO (~60%), MAC+AXOCLAV (~40%), MAC+PIME (~40%) and MAC+MERO (~20%). The observed data provide an experimental baseline reference for our phenotypic quantification methodology. Upon incubation of the starting 10-strain (5-gene groups x 2 strains per group) bacterial consortium for 24 hours in plain CAMH-2 broth, the relative proportions of CFU on the selective antibiotic plates were: MAC+TET plate (~ 105%; note, likely a counting artifact), the MAC+AMP plate (~ 27.7%), MAC+FOX (~7.2%), MAC+AXO (~23.1%), MAC+AXOCLAV (~10.6%), MAC+PIME (~4.4%) and MAC+MERO (~0.05%) (see Table 4).

**Table 4:** Within-column relative quantities (%) of estimated bacterial counts from various antibiotic broth concentrations subsequently grown on plain versus selective MacConkey agar plates.

MacConkey plate type	Input Mixture (no broth incubation)	Plain CAMH-2 broth (0 μg/mL)	Ampicillin CAMH-2 broth (2 μg/mL)	Ampicillin CAMH-2 broth (4 μg/mL)	Ampicillin CAMH-2 broth (8 μg/mL)	Ampicillin CAMH-2 broth (16 μg/mL)	Ampicillin CAMH-2 broth (32 μg/mL)	Ceftiofur CAMH-2 broth (0.5 μg/mL)	Ceftiofur CAMH-2 broth (1 μg/mL)	Ceftiofur CAMH-2 broth (2 μg/mL)	Ceftiofur CAMH-2 broth (4 μg/mL)	Ceftiofur CAMH-2 broth (8 μg/mL)
	Column % Log_10_ CFU	Column % Log_10_ CFU	Column % Log_10_ CFU	Column % Log_10_ CFU	Column % Log_10_ CFU	Column % Log_10_ CFU	Column % Log_10_ CFU	Column % Log_10_ CFU	Column % Log_10_ CFU	Column % Log_10_ CFU	Column % Log_10_ CFU	Column % Log_10_ CFU
**MAC**	**100**	**100**	**100**	**100**	**100**	**100**	**100**	**100**	**100**	**100**	**100**	**100**
	**7.16**	**9.58**	**9.60**	**9.55**	**9.32**	**9.25**	**9.32**	**9.17**	**9.24**	**9.11**	**8.87**	**8.93**
**MAC+TET**	91.48	105.59	85.89	94.37	92.68	94.80	82.28	94.12	80.31	69.59	52.50	59.69
**MAC+AMP**	95.34	27.66	29.11	34.72	59.15	100.37	89.87	115.84	108.11	98.45	86.67	107.75
**MAC+FOX**	19.36	7.18	6.81	5.93	28.05	40.52	27.85	43.44	37.45	64.43	70.83	86.82
**MAC+AXO**	64.73	23.14	27.50	30.53	51.52	96.28	86.71	120.36	98.07	86.08	94.17	103.10
**MAC+AXOCLAV**	37.80	10.64	6.77	5.76	23.78	43.87	48.73	56.56	52.90	60.31	78.33	108.53
**MAC+PIME**	14.69	4.43	3.63	4.71	10.37	14.13	13.29	28.96	11.58	41.24	65.83	103.88
**MAC+MERO**	10.71	0.05	0.04	0.02	0.10	0.16	0.13	0.15	0.10	0.30	0.82	1.92

MAC (Plain MacConkey), +TET (Tetracycline—16 μg/mL), +AMP (Ampicillin—32 μg/mL), +FOX (Cefoxitin—32 μg/mL), +AXO (Ceftriaxone—4 μg/mL), +AXOCLAV (Ceftriaxone—4 μg/mL and clavulanate -4 μg/mL), +PIME (Cefepime—8 μg/mL) and +MERO (Meropenem -4 μg/mL and 1 μg/mL); CAMH-2 (Cation-adjusted Mueller Hinton II); CFU (Colony forming unit).

#### Ampicillin

No significant difference was observed (Table 4) between the estimated (via plain MacConkey agar plates) total bacteria CFU in the mixed population culture after 24-hour incubation in non-selective CAMH-2 broth [9.58 (log_**10**_CFU/mL) 95% CI: 9.44–9.72] compared to the lowest ampicillin broth concentration (2 μg/mL) that we tested [9.60 (log_**10**_CFU/mL) 95% CI: 9.50–9.69]. In direct contrast to an increasing concentration of ampicillin, a steady decrease in estimated total bacteria population CFU in the mixed-strain culture was observed. Across the tested concentration range, estimated bacterial density decreased from 9.60 (log_**10**_CFU/mL) [95% CI: 9.50–9.69] to 9.32 (log_**10**_CFU/mL) [95% CI: 9.21–9.43]. The magnitude of reduction in estimated total CFU (relative to total CFU in plain broth, the maximum carrying capacity) was largest (40.5%), between ampicillin broth concentrations of 4 μg/mL and 8 μg/mL.

**Fig 4:**
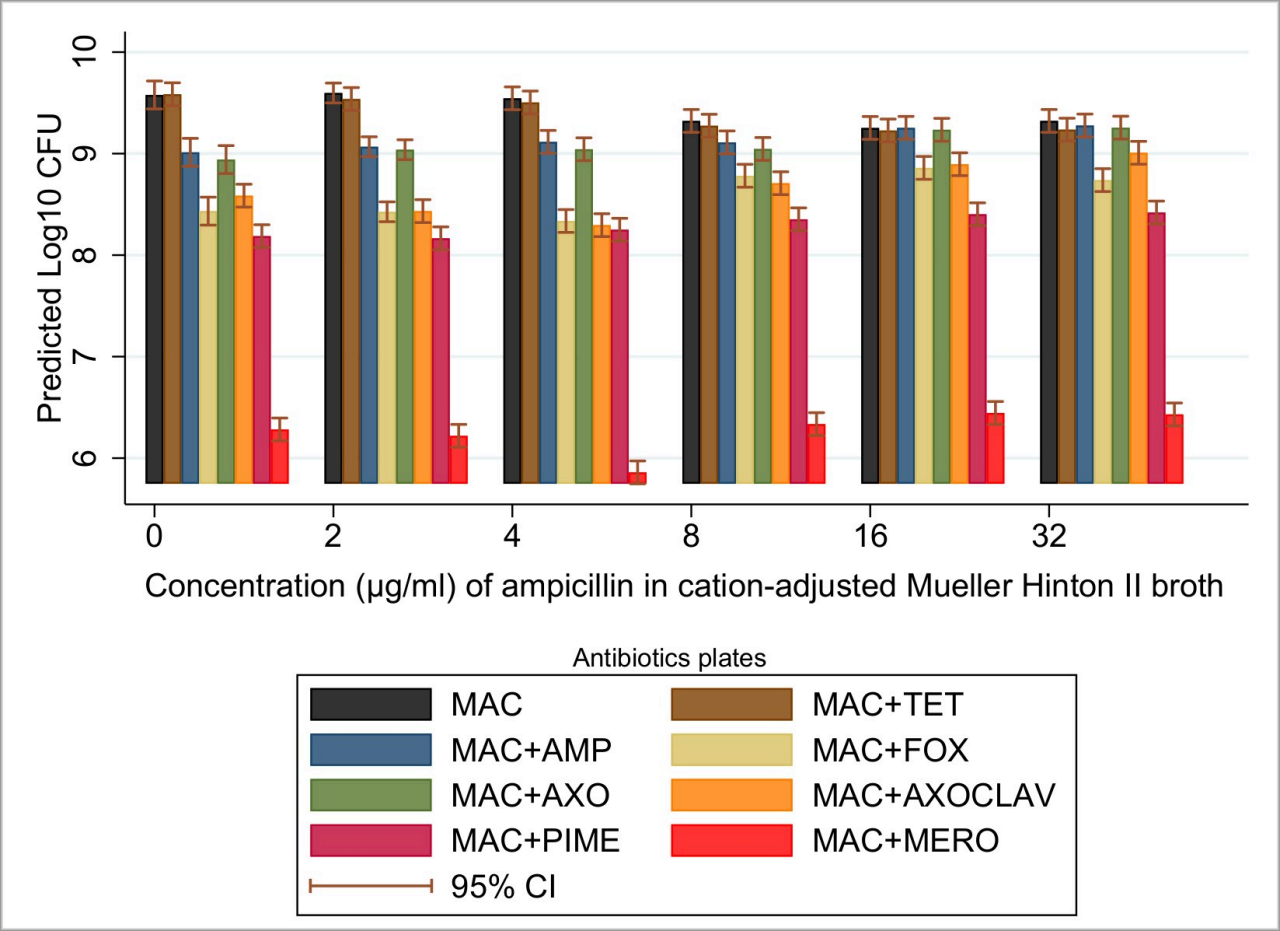
Effects of varying ampicillin concentrations on 10-strain mixed-bacterial culture. Predicted marginal mean counts (log_10_CFU) with 95% CI following 24-hour incubation in ampicillin (at 2, 4, 8, 16, 32 μg/mL) in cation-adjusted Mueller-Hinton II broth. *The selective MacConkey agar plate antibiotic concentrations were: ampicillin (32 μg/mL), tetracycline (16 μg/mL), cefoxitin (32 μg/mL), ceftriaxone (4 μg/mL), ceftriaxone (4 μg/mL) + clavulanic acid (4 μg/mL), cefepime (8 μg/mL), and meropenem (4 μg/mL); CFU (Colony forming unit).

The constituent proportions of beta-lactamase producers in the ampicillin containing cultures, as estimated by the CFU counts on the MAC+AMP agar plates relative to the non-selective plate, were 29%, 34.7%, 59.1%, and 100% for 2, 4, 8, and 16 μg/mL, respectively, in ascending order of ampicillin concentration. In contrast to this trend, the beta-lactamase producers were estimated to grow only to 90% of the total expected CFU in the 32 μg/mL ampicillin broth culture, a substantial drop from the preceding concentration. Nonetheless, the absolute count of the beta-lactamase producers increased steadily across ampicillin concentrations from 9.07 (log_10_CFU/mL) [95% CI: 8.97–9.17] in the 2 μg/mL ampicillin broth culture to 9.28 (log_10_CFU/mL) [95% CI: 9.16–9.39] in the 32 μg/mL ampicillin broth culture. Although this result exhibits a seeming paradoxical increase in relative proportions of non-beta-lactamase producers in 32 μg/mL ampicillin broth culture, the overall trend observed was a steep decline in the susceptible bacteria sub-population and a steady increase in the component beta-lactamase producing strains’ population as *in vitro* ampicillin concentrations increased. The estimated proportion of CPE strains in the mixed culture quadrupled from the 2 μg/mL ampicillin broth to the 16 μg/mL ampicillin broth, increasing from 0.04% of the estimated total density to 0.16% (peak). Bacterial counts on the MAC+AXOCLAV plates also generally increased in proportion along with ampicillin broth concentration: 6.8%, 5.8%, 23.8%, 43.9% and 48.7% (for 2, 4, 8, 16, and 32 μg/mL, respectively). Likewise, bacterial count estimates on the MAC+AXO plates increased in proportion with doubling ampicillin concentrations, peaking at ampicillin concentrations of 16 μg/mL: 27.5%, 30.5%, 51.5%, 96.3% and 86.7%, and in ascending order of ampicillin concentration. The marginal mean estimates from the MAC+AXO plates, MAC+AXOCLAV plates and the MAC+AMP plates suggest the ESBL-producing strains constitute the bulk of beta-lactamase producers following selective pressures of ampicillin in a competitive mixed culture (see Table 4 and Fig 4).

#### Ceftiofur

In contrast to the starting ampicillin broth concentration (2 μg/mL), the starting ceftiofur broth concentration (0.5 μg/mL) demonstrated a significant suppression of the estimated total bacterial count relative to estimates from the non-selective broth culture [9.17 (log_**10**_CFU/mL) 95% CI: 9.08–9.26 compared to 9.58 (log_**10**_CFU/mL) 95% CI: 9.44–9.72], respectively (Table 4). Further significant (P < 0.05) suppressions of the estimated total bacterial CFU in the mixed-strain culture were not observed until the broth concentration doubled from 2 μg/mL to 4 μg/mL of ceftiofur [9.11 (log_**10**_CFU/mL) 95% CI: 9.02–9.20 compared to 8.87 (log_**10**_CFU/mL) 95% CI: 8.78–8.96], respectively. The proportion of the maximum capacity (that is, in absence of the antibiotic) achieved by beta-lactamase producers in the ceftiofur broth culture (as estimated by the CFU counts on the MAC+AMP agar plates relative to the non-selective plate) were 115%, 108%, 98%, 87% and 108%, in ascending order of ceftiofur concentration. The proportions above or near 100% suggest dominance of the culture by such strains and are likely artifacts introduced by bacterial counting methods when they exceed 100% of growth on plain media.

**Fig 5:**
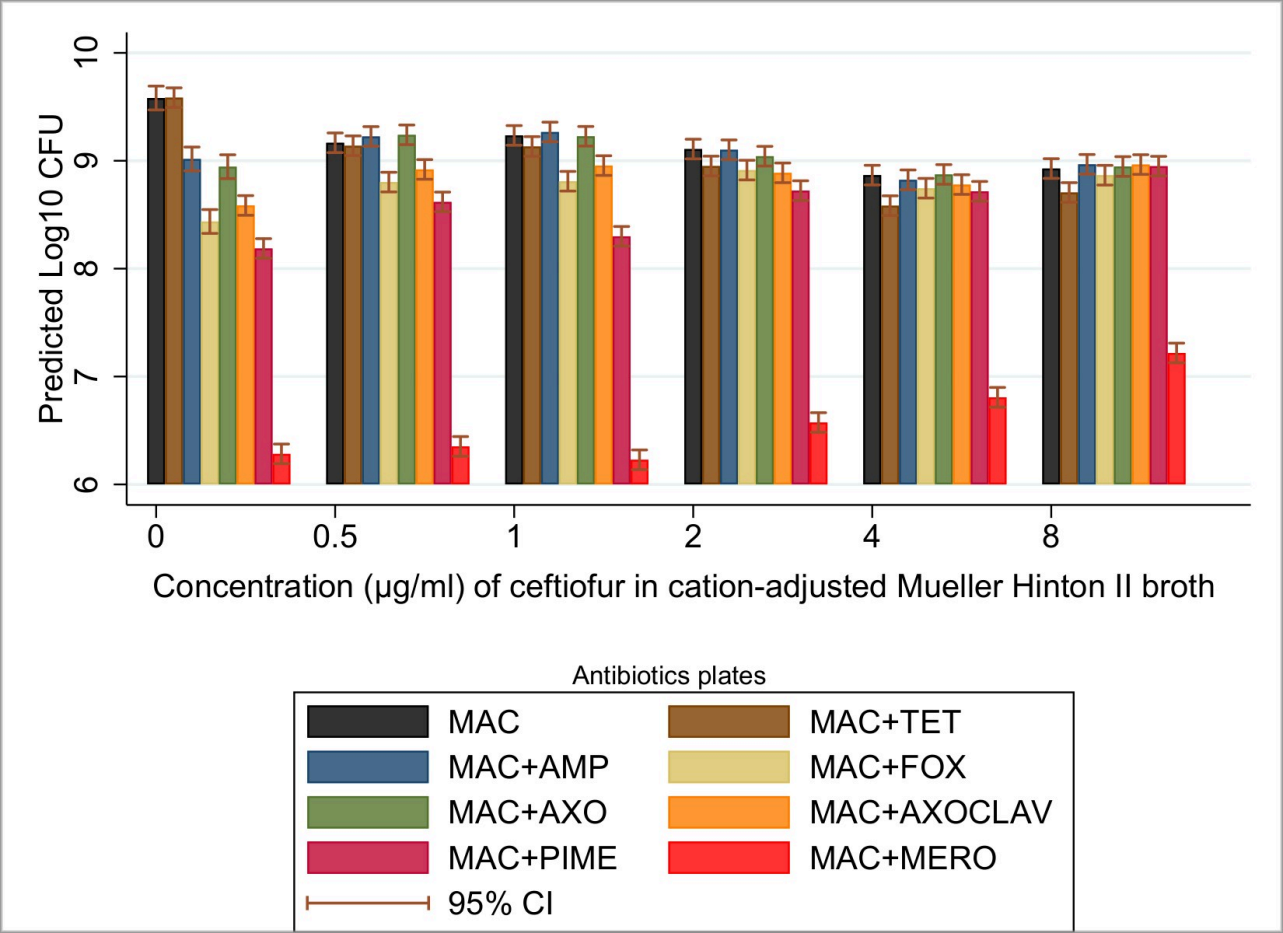
Concentration effects of ceftiofur on 10-strain mixed-bacterial culture. Predicted marginal mean CFU with 95% CI following 24-hour incubation in ceftiofur (at 0.5, 1, 2, 4, 8 μg/mL) in cation-adjusted Mueller-Hinton II broth. *MacConkey agar plate antibiotic concentrations: ampicillin (32 μg/mL), tetracycline (16 μg/mL), cefoxitin (32 μg/mL), ceftriaxone (4 μg/mL), ceftriaxone (4 μg/mL) + clavulanic acid (4 μg/mL), cefepime (8 μg/mL), and meropenem (4 μg/mL); CFU (Colony forming unit).

The absolute count of the beta-lactamase producers as estimated on these plates also decreased; most notably, they were suppressed in the broths with 4 μg/mL and 8 μg/mL of ceftiofur. The overlap in estimated CFU proportions on the MAC+AMP and MAC+AXO selective agar plates suggests the TEM-1-type beta-lactamase strains constituted a less important component of the community. The estimated proportion of CPE strains isolated via the MAC+MERO selective media plates showed a steady increase from 0.2%, through 0.1%, 0.3%, 0.8%, and 1.9%, along the ceftiofur concentration gradient outlined in Table 1. The CFUs selected by the MAC+AXOCLAV plates also increased in proportion along with ceftiofur concentrations: 56.6%, 52.9%, 60.3%, 78.3% and 108.5% (see Table 4 and Fig 5).

## Discussion

Often, and for good reasons, regulatory and policy approaches to the challenge of AMR tend to be reactive rather than proactive. These are exemplified by restrictions on the use of specific antimicrobial drugs, classes, or drug indications for specific diseases, often long after they have been approved and labeled for such use. The current absence of carbapenems and 4GC use in the U.S. in livestock production has offered unique opportunities for assessing the value of proactive policies to addressing the question of the roles of direct and indirect selection of bacterial resistance types to these antimicrobials in the presence and absence of co-selection by other antimicrobials.

Though more consistently seen with chromosomally located genes, the carriage of AMR genes on horizontally transferable genetic materials has also been shown to impose a fitness cost (often estimated using the maximal growth rate as a surrogate fitness measure) on bacterial strains [75,76]. In our study, mammalian-derived commensal *E*. *coli* strains with similar beta-lactamase genotypes were found to possess a spectrum of fitness levels as reflected in the growth rates. This underscores the importance of a global genotypic evaluation across all AMR profiles, virulence profiles and other stress adaptation mechanisms, in order to estimate the fitness impact of a particular variable. In this study, unlike in prior studies [77], we characterized the growth of a large number of bacterial strains for each gene group to assess and adjust for this variation among strains of the same species exhibiting similar genotypic and phenotypic characteristics. When analyzed based on the *bla-*gene classifications (n = 20 per gene group), our study found the *bla-*negative group to be the most fit in the absence of antibiotic selection pressures, suggesting a fitness advantage may exist in association with the lack of a *bla* gene. A similar model comparing beta-lactamase producers, surprisingly, showed a similar level of fitness between the high-potency carbapenemase *bla* gene group and the lower potency *bla* gene group (*bla*_TEM-1_). Consequently, our results do not suggest a trend of increasing fitness cost (as estimated by the maximum growth rate) in lock step with increasing resistance severity of the *bla* gene groups. On the other hand, a relatively impaired maximum bacterial density (as implied by the highest OD as a surrogate measure) was observed for the carbapenemase-producing group; taken together, this may constitute a more important indicator of relative fitness cost in an enteric environment with limited nutrient-based carrying capacity and intense competition with other strains utilizing the same resource. Diminished group total bacterial density estimates in the antibiotic-supplemented media may indicate a different form of impaired bacterial fitness that could also impact population dynamics. Indeed, the biphasic exponential growth phase and the prolong lag phase duration seen in raw data plots (Fig 1) were not reflected in the model predictions nor the fitted plots of the growth curves respectively.

When bacteria of mixed origin are in a community, competition is known to occur, either due to limitations in resource availability or else as strains adapt and obtain an evolutionary advantage [78,79]. For instance, Ushijima and Seto demonstrated in an *in vitro* study that a group of normal intestinal commensals–*E*. *coli*, *E*. *aerogenes*, *Enterococcus faecalis*, *Bacteroides ovatus* and *Fusobacterium varium*–are capable of suppressing the growth of an intestinal pathogen–*Salmonella* Typhimurium–under certain conditions [80]. In addition, bacterial ecologists have noted that when members of a bacterial community are genetically similar, antagonism/inhibition is less likely to occur; in contrast, cooperation may ensue [81]. Given the similarity (same species) of our limited number of study strains, it is reasonable to expect that the fitness differential would predict the population dynamics of the bacteria community, especially given the similar profile of nutrient requirements and ecological niche. The fitness pattern of the five sets of representative pairs of strains from the five *bla*-gene-based groups in the mixed populations followed an overall similar trend to the estimates with twenty strains per group, each grown as mono-culture (Figs 1 and 2). That said, the margins of fitness advantage exhibited by the *bla-*negative pair and the pair of carbapenemase producers over the other pairs chosen for the competitive assay were slightly more pronounced (Tables 2 and 3). Our results showed a preponderance of the *bla-*negative strains in the bacterial community after 24-hour incubation in broth without a selection pressure, an expected outcome in the absence of mutual growth interference by the component *E*. *coli* strains. The data also showed a seeming suppression of the carbapenemase-producers beyond the limitations conferred by their growth rates; however, this may instead reflect CPE density under-estimation using the agar plates supplemented with 4 μg/mL meropenem (and perhaps point to a need for additional micro-nutrient supplementation such as zinc). Negri et al., in their *in vitro* mixed culture study of beta-lactam resistant and susceptible *Streptococcus pneumoniae*, similarly found no interference among *S*. *pneumoniae* strains with different profiles of resistance to beta-lactams [77].

Mollenkopf et al., in their CPE surveillance study of a swine production facility, found a 16.5% detection risk across environmental and fecal samples [49]. Notably, virtually all (~100%) of their CPE (*bla*_IMP-64_/IncQ1) positive samples originated from the farrowing barns; importantly, the authors attributed this finding to the use of ceftiofur in the sows (on-label treatments as needed) and in the piglets (extra-label use for infection prevention and control such as following castration of male piglets) [49]. As mentioned earlier, when estimated across twenty strains, the carbapenemase-producers did not appear to exhibit a high fitness cost relative to other *bla-*positive strains; importantly, this suggests that even absent beta-lactam selection pressure and with a sufficiently high initial population, CPEs could persist at a low prevalence along with other beta-lactam-resistant Enterobacteriaceae. Instead, the relatively lower maximum OD of the CPE cultures may represent the real factor associated with lower levels of CPE currently found in food animal production environments.

The introduction into the bacterial growth media of different generations of beta-lactam antibiotics and at increasing concentrations was observed to change the population dynamics of the *in vitro* bacterial consortium. Increasing concentrations of ampicillin sodium gradually inhibited the *bla-*negative sub-population while selecting for the beta-lactamase resistant strains, including the full spectrum from TEM-1-type beta-lactamase producers through CPE strains. In contrast, low concentrations of ceftiofur hydrochloride (0.5 μg/mL) completely suppressed the susceptible populations of *bla-*negative and TEM-1-type beta-lactamase producers, thus effectively selecting for the CMY-2, CTX-M, and CPE resistant sub-populations. Increasing concentrations of ceftiofur hydrochloride suppressed the total bacterial density while further increasing the proportions of the higher-level beta-lactamase producing strains; for example, the carbapenemase producers showed about a forty-fold jump in relative proportion from non-selective broth through to 8 μg/mL of ceftiofur hydrochloride broth. Our results show that a 3^rd^ generation cephalosporin (3GC) such as ceftiofur provides a more than adequate selection advantage for carbapenemase producers, even in the absence of direct selection (i.e., carbapenem use) and minimal indirect co-selection. The presence of co-selected *bla* genes on the same plasmids could further aggravate the selection observed, given the carriage of many plasmid borne resistance genes has been observed to impose little or no additional fitness cost to the organism [82,83]. In our own study, it was difficult to find strains harboring either AmpC or ESBL genes that did not also harbor *bla*_TEM-1_. Among the CPE strains, it was even more difficult to find strains that lacked not only *bla*_TEM-1_, but also AmpC or ESBL genes. Genes are generally added to an existing arsenal of resistance and virulence factors in the strains. These findings agree with the observations in the surveillance study of the swine production facility by Mollenkopf et al. that ceftiofur provided adequate selection advantage for carbapenemase-producers to emerge into detectable levels [49].

In both ampicillin- and ceftiofur-supplemented CAMH-2 broth, the ESBL sub-population appeared to increase in dominance with increasing antimicrobial concentrations. This was likely facilitated by their relatively high minimum inhibitory concentrations (MIC) for these antimicrobials [84] and their fitness advantage over other *bla-*positive strains (Table 3). Overall, our findings among Enterobacteriaceae are remarkably similar to those of Negri et al.in gram-positive bacteria, which tested the effect of varying concentrations of amoxicillin, cefixime, cefuroxime and cefotaxime on a mixture of *S*. *pneumoniae* strains with MIC values ranging from susceptible to resistant across the antimicrobial agents. The newer generation beta-lactams completely suppressed the susceptible strains while selecting for higher resistance strains; in contrast, the less potent amoxicillin, at lower concentrations, mildly suppressed the susceptible strains while selecting effectively for the low-level beta-lactamase producing strains [77]. Ambler class C beta-lactamase enzymes, such as encoded by the plasmid-borne *bla*_CMY-2_ gene and the Ambler class A enzyme encoded by the plasmid-borne *bla*_CTX-M_ gene have both been determined to be effective against 3GC antibiotics [26,84]. However, in 4 μg/mL ceftriaxone broth the CMY-2 type beta-lactamase strains demonstrated impaired group growth rates compared to the CTX-M* type beta-lactamase strains (Table 2 and Fig 3). This observed difference in their growth potential suggests that with any given mixed bacterial community exposed to a similar or higher concentration of ceftriaxone, the strains harboring the *bla*_CTX-M_ genes would be favored over the CMY-2 type beta-lactamase producing strains. Despite this theoretical outcome, in our mixed-strain cultures grown in ceftiofur broth, the relative proportion of the AmpC-type *bla* strains continued to increase along with the concentration of ceftiofur all the way to the highest experimental concentration. This occurred, rather than a plateauing or reduction in the relative proportion of these strains at the highest ceftiofur concentrations.

In the 10-strain competition assay, there was a preponderance of susceptible strains in the bacterial community in the absence of antibiotics, when compared with the beta-lactamase producers, as would be expected giving the superior fitness of the susceptible strains (Table 3). This is also consistent with contemporary estimates of beta-lactam resistance prevalence among indicator organisms such as *E*. *coli*, especially absent antibiotic selection-pressure. In contrast to estimated fitness values, the proportion of carbapenemase-producers, as measured by way of the MAC+MERO plates, was not comparable with the AmpC-type beta-lactamase strains. It should be noted that the component CPE population was estimated at 1 μg/mL meropenem (MAC+MERO) agar plates for the starting mixture and 4 μg/mL meropenem (MAC+MERO) agar plates for the post-incubation mixture, due to serious strain inhibition below the limits of detection at the higher meropenem concentration for the starting mixture; consequently, the CPE strain population was likely to be underestimated at the higher meropenem concentration. However, as the resistance breakpoint established by CLSI [46] is at 4 μg/mL meropenem (MAC+MERO) this was how the study was designed.

Importantly, the limitations inherent in models such as employed in this research must be considered in evaluating their usefulness. For instance, it should be noted that in nature the starting populations of bacteria with various resistance profiles are unlikely to be equal as was modeled in this study; therefore, the post-exposure changes in the prevalence of the resistant bacterial strains may not be as remarkable as we determined. Furthermore, *in vivo* antimicrobial concentrations are not constant, as was the case with our *in vitro* model; rather, the drug and active drug metabolite concentrations dynamically rise and fall, based on the dosing regimen and the drug distribution, metabolism and excretion. Still, recommended antimicrobial regimens are known to expand resistant coliform populations in livestock intestinal flora, sometimes for several weeks after the final drug administration [14]. Also, in nature, a distinct separation of bacterial groups by beta-lactamase enzyme profile is unlikely; that is, resistant bacteria frequently harbor multiple resistance determinants against a single class of antimicrobial agent, as well as to different classes of agents. Overlapping sets of resistance genes, as was frequently encountered during our isolate selection for this study, would be expected to add layers of complexity to the selection dynamics in nature. Although, our strain-group sample size was limited to twenty strains each (due to restricted availability), instead of thirty strains as projected by statistical theorem; Gompertz-3 model estimated standard errors of growth parameters were however very narrow, hence suggesting a rather robust estimate with the employed sample size. Consequently, we do not expect the strain-group sample size to negatively impact the parameter estimates. Similarly, due to availability constraint, several members of the carbapenemase producing strain-group bore supplementary *bla*-type genes. Given that the selection process for these strains (CPEs) are occurring in hospital environments rather than in the community where pan susceptible strains are more prevalent, their carriage of multiple resistance genes seems unavoidable due to the cumulative addition of novel genes to an existing array of resistance genes rather than *de novo* acquisition by susceptible strains. These may surely cloud the interpretation of the reported results, in particular the group-fitness estimates, and potential co-selection effects by test antimicrobials. In our mixed-strain experiments however, only a member of the pair of CPE representative strains bore a add-on bla gene (*bla*-_CMY-2_); we importantly note that, our data did not indicate a relative fitness cost or an amplified co-selection of these representative strains.

Our study did not factor in the role of innate bacteria resistance mechanisms, such as non-specific efflux pumps and membrane-porin down regulation [85], because the presence of such mechanisms can be expected to exert a relatively uniform non-specific effect across study strains; however, for beta-lactam antibiotics this is not likely to interfere with interpretations since most resistance is enzymatic and the damage inflicted by the antibiotic is to the cell wall. Lastly, the influence of far more abundant and niche-competing anaerobic intestinal commensals, or free-living environmental strains, in antimicrobial selection was not modeled in this study. Although the conditions of these *in vitro* models may not perfectly approximate *in vivo* or environmental realities, they nonetheless constitute a reasonable first step in a systematic and order approach to this challenge.

Overall, our hypothesis that older generation beta-lactam antibiotics of lesser priority–such as ampicillin–can also provide a selection advantage to highest priority resistance types–such as 3rd/4th generation cephalosporins and carbapenems–albeit less efficiently when compared to 3^rd^ generation cephalosporins, was supported by the results of these two *in vitro* experimental studies. Currently, beta-lactam antimicrobial resistance due to carbapenemase producing Enterobacteriaceae is not a known existential therapeutic threat in animal agriculture; in contrast, human health care infections caused by the *K*. *pneumoniae* producing KPC-type *bla* enzyme are a global concern [1]. These strains, along with other Enterobacteriaceae bearing less prevalent carbapenemase encoding genes (e.g., *bla*_NDM_, *bla*_VIM_ & *bla*_IMP_) have been reported in many U.S. states [16]. Presently, the challenge posed by carbapenemase-producing bacteria is limited to specific settings in the human healthcare system. Although *E*. *coli* strains with carbapenemase-encoding genes (including the *bla*_KPC_ gene) have been identified [86,87], community acquired infections with strains bearing this order of beta-lactam resistance are still relatively rare [88].

Our study suggests that if introduced into food-animal populations, perhaps through surface water downstream from hospital and wastewater treatment plant effluent discharge [89], CPEs along with other high priority beta-lactamase resistance profiles could be expanded due to lower-priority beta-lactam use in food animals, and subsequently spread back to the human community through food-animal products and via the livestock environment. To further clarify and characterize these findings, we suggest additional studies, both observational and experimental. For example, an *in vitro* continuous anaerobic medium such as employed by Ushijima and Seto might provide a better model of the selection dynamics expected in a monogastric mammalian bowel compared to the 10-strain batch culture we used [80]. Further, pharmacokinetics-pharmacodynamics (PK-PD) mathematical modeling of these selection dynamics and ultimately *in vivo* animal studies would be logical next steps.

In conclusion, this study showed that the absence of direct carbapenem selection pressure in food-animal production cannot be relied upon alone to reduce the spread of bacterial strains with reduced carbapenem susceptibility. Use of commonly prescribed older-generation beta-lactams such as ampicillin and ceftiofur can expand both ESBL Enterobacteriaceae and CPE prevalence in commensal and pathogenic bacterial communities.

## Supporting information

S1 Table *E*. *coli* (n = 100) sequencing data and phenotype results. (XLSX)
